# Increasing Genetic Variance of Body Mass Index during the Swedish Obesity Epidemic

**DOI:** 10.1371/journal.pone.0027135

**Published:** 2011-11-07

**Authors:** Benjamin Rokholm, Karri Silventoinen, Per Tynelius, Michael Gamborg, Thorkild I. A. Sørensen, Finn Rasmussen

**Affiliations:** 1 Institute of Preventive Medicine, Copenhagen University Hospital, Centre for Health and Society, Copenhagen, Denmark; 2 Population Research Unit, Department of Social Research, University of Helsinki, Helsinki, Finland; 3 Department of Public Health Sciences, Karolinska Institutet, Stockholm, Sweden; Ohio State University Medical Center, United States of America

## Abstract

**Background and Objectives:**

There is no doubt that the dramatic worldwide increase in obesity prevalence is due to changes in environmental factors. However, twin and family studies suggest that genetic differences are responsible for the major part of the variation in adiposity within populations. Recent studies show that the genetic effects on body mass index (BMI) may be stronger when combined with presumed risk factors for obesity. We tested the hypothesis that the genetic variance of BMI has increased during the obesity epidemic.

**Methods:**

The data comprised height and weight measurements of 1,474,065 Swedish conscripts at age 18–19 y born between 1951 and 1983. The data were linked to the Swedish Multi-Generation Register and the Swedish Twin Register from which 264,796 full-brother pairs, 1,736 monozygotic (MZ) and 1,961 dizygotic (DZ) twin pairs were identified. The twin pairs were analysed to identify the most parsimonious model for the genetic and environmental contribution to BMI variance. The full-brother pairs were subsequently divided into subgroups by year of birth to investigate trends in the genetic variance of BMI.

**Results:**

The twin analysis showed that BMI variation could be explained by additive genetic and environmental factors not shared by co-twins. On the basis of the analyses of the full-siblings, the additive genetic variance of BMI increased from 4.3 [95% CI 4.04–4.53] to 7.9 [95% CI 7.28–8.54] within the study period, as did the unique environmental variance, which increased from 1.4 [95% CI 1.32–1.48] to 2.0 [95% CI 1.89–2.22]. The BMI heritability increased from 75% to 78.8%.

**Conclusion:**

The results confirm the hypothesis that the additive genetic variance of BMI has increased strongly during the obesity epidemic. This suggests that the obesogenic environment has enhanced the influence of adiposity related genes.

## Introduction

The prevalence of obesity has increased strongly in Sweden during the last decades, and 10% of all Swedish men and women are now obese [1], [2] In the USA the prevalence of obesity has reached epidemic proportions and recent data show that more than a third of all men and women are obese [3], [4].

There is no doubt that environmental factors have initiated the epidemic, since the gene pool in the population changes at a rate that is much too slow to explain the observed pattern. Nevertheless with heritability (the proportion of phenotypic variation attributable to genetic differences among individuals) of body mass index (BMI) in the range of 50–80%, twin studies suggest that genetic differences account for the majority of variation in body fatness within populations [5], [6]. Furthermore, there is evidence for a continuously high, and possibly increasing, heritability of BMI despite the increasing effect of environmental factors, which gave rise to the obesity epidemic [7]. This seems paradoxical since environmental changes have resulted in an increase in the total BMI variation [8]. An explanation could be that the genetic influence on BMI has increased because of the influence from the obesogenic environment, responsible for the increase in the prevalence of obesity. Such an association suggests that the effect of genes depends upon the environmental exposure, which implies gene-environment interaction (GxE).

So far various presumed environmental risk factors for obesity have been investigated as potential effect modifiers of genetic effects on adiposity. Twin studies conducted in several populations have found lower heritability of obesity in physically more active individuals [9]–[11]. Similarly on a molecular genetic level, several studies have recently found that physical activity attenuates the effect of the fat mass and obesity associated (FTO) gene and other genetic loci that are associated with body fatness [12]–[15]. In addition, fat and carbohydrate intake has been found to interact with the *FTO* gene on BMI [16].

These results all suggest that genetic effects on adiposity are modifiable by environmental influences, which are related to the obesogenic environment. However, the research is still at an early stage and it is possible that the discovered interactions apply to other genetic loci and to other environmental factors than physical activity and fat and carbohydrate intake. On this background we tested the more general hypothesis that the genetic variance of BMI has increased during the obesity epidemic among Swedish young men. If the hypothesis is confirmed it may imply that the obesogenic environment reinforces the effect of genes related to adiposity. Although a similar hypothesis has been investigated previously on Danish twin data [17] the current study is the first to investigate an actual secular trend in the genetic variance of BMI.

## Methods

### Ethics statement

This study has been ethically approved by the Swedish ethical approval system (Centrala etikprövningsnämnden, Vetenskapsrådet i Stockholm). Informed consent was not considered necessary by the Swedish ethical approval system since the study relies purely on non-identifiable register-based data.

### Study population

The data comprises height and weight measurements of all Swedish men born between 1951 and 1983 who underwent military conscription examination. Height was measured using a wall-mounted stadiometer and weight using an analogue or digital scale. BMI was calculated as weight (kg)/height^2^ (m^2^). Extreme BMI values (15 kg/m^2^>BMI>50 kg/m^2^) were excluded to reduce the risk of misclassification due to measurement or data entry errors. During the years covered by this study, conscription examination was compulsory by law for all young men with Swedish citizenship. Only men with severe diseases and disabilities were exempted based on a certificate issued by a physician with information on diagnosis.

Information on sibling status was acquired by linking the conscript data with the Swedish Multi-Generation Register and the Swedish Twin Register using the Swedish personal identification number (ID) unique to each subject. The ID numbers of the biological parents were included in the record of their offspring and on this background 264,796 pairs of full-brothers and 1,736 MZ and 1,961 DZ twin pairs were identified among the conscripts. Data on zygosity were obtained from the Swedish Twin Register and from the Swedish Young Male Twins Study [18], [19]. Due to lack of information on zygosity 2,452 twin pairs were excluded. To limit the possible change in childhood family environment, brothers born more than 3 years apart were excluded resulting in a total of 116,478 brother pairs for analysis (Table 1).

**Table 1:** Summary statistics for BMI in twins and full-brothers.

	N	Mean	Variance	Covariance [95%CI]	Correlation [95%CI]
Full-brothers	116,478	21.69	7.88	2.96 [2.94–2.98]	0.37 [0.37–0.38]
DZ twins	1,961	21.05	5.65	2.33 [2.19–2.48]	0.43 [0.39–0.47]
MZ twins	1,736	21.04	5.61	4.72 [4.42–5.05]	0.83 [0.82–0.85]

### Statistical analyses

The sibling pairs were analysed through quantitative genetic modelling, in which the total phenotypic variance of a trait is partitioned into environmental and genetic components. The disentangling of genetic and environmental variance is made possible through structural equation modelling of covariance within pairs of different types of family relations. The relations included in the modelling are genetically informative if they differ in the degree of genetic or environmental similarity [20]. In the case of MZ and DZ twin pairs, the genetic variance can be estimated since MZ twins have the same gene sequence, while DZ twins share, on average, 50% of their segregating genes. This, however, rests on the assumptions that the common environmental influences are the same for MZ and DZ pairs. Furthermore, this study design requires equal phenotype means and variances for MZ and DZ pairs.

The genetic variance can further be decomposed into parts due to additive genetic effects (A) and dominance genetic effects, i.e. interaction between alleles on the same locus, (D) over all relevant loci. Epistatic effects refer to interaction between different loci. If the loci are unlinked, e.g. by being on different chromosomes, these effects are modelled as part of dominance genetic effects. In the case of linked loci, epistatic effects will be modelled as additive genetic effects since the loci segregate together and form a unit. The environmental variance can be divided into factors shared by co-twins (C) and factors that are unique to each twin individual including also any measurement error (E). Hence, the total phenotypic variance can be decomposed into four components: the additive genetic (A), dominant genetic (D), common environmental (C), and unique environmental (E) variance component.

BMI was used as a measure of body fatness. Two sets of statistical analyses were conducted to calculate the variance components in BMI. The first is a classical twin analysis using MZ and DZ twin pairs [20] from which we identified the most parsimonious variance components model for BMI. The twin analyses in the current study were conducted using the Mx statistical software, version 1.7.03 [21]. Mx derives structural equation models from twin and family data and calculates the variance components through maximum likelihood estimation. Since we only have data on twins reared together, the D and C components could not be estimated simultaneously, and thus the model fit of the ACE and ADE models were compared. Subsequently, the significance of each variance component was tested and the most parsimonious model was selected.

Assuming that the AE model adequately explains BMI variance, the second set of analyses investigated the secular trends in the A and E components using only full-brother pairs. The twin study complements the full-brother pair analysis by testing whether this assumption is reasonable. The advantage of using full brother pairs in this second phase is that this data set is considerably larger which improves the statistical power to detect possible secular changes. The brother pairs were divided into birth cohorts defined by the birth year of a randomly selected brother in a pair. The BMI variance and brother pair covariance was then calculated for each birth cohort. Assuming the absence of common environmental and dominant genetic influences, or assuming that these influences are very small, the covariance multiplied by two is an estimate of the additive genetic variance in BMI since brothers share, on average, half of their segregating genes such as DZ twins. The remaining variance can be attributed to the non-shared environmental influences. A sensitivity analysis approach was used to investigate whether the same results could be obtained when normalising the BMI distribution with the logarithmic transformation.

To compare the development in the variance components with the general course of the Swedish obesity epidemic, we calculated obesity prevalence for each birth year using the WHO defined cut-point of BMI≥30 [22]. Pearson correlation coefficients for the relationship between prevalence of obesity and the A and E variance components were calculated. The purpose was to explore the hypothesis that changes in the variance components are related to the influence of the obesogenic environment. For higher accuracy of the estimates, we used the whole sample of conscripts in the calculation of prevalence estimates.

## Results

**Figure 1:**
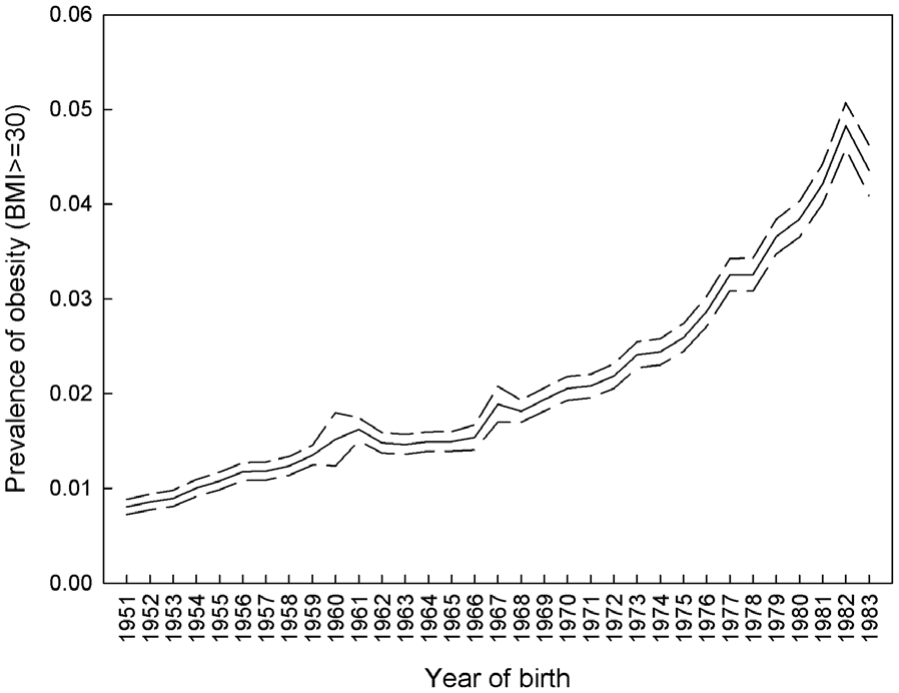
The prevalence of obesity in the full sample of conscripts by year of birth with 95% CI.

The prevalence of obesity increased more than five-fold from 0.8% to 4.4% from the 1951 to the 1983 birth cohort (Figure 1). Less strong increase over time in prevalence of overweight and even stronger increase in prevalence of severe obesity has been reported previously [1]. Two phases can be identified from the development. The first phase includes the birth cohorts from 1951 to around 1973 where the increase in prevalence was moderate. In the second phase the increase became significantly stronger with the birth cohorts from around 1973 and onward.

Table 1 shows summary statistics for full-brother pairs and MZ and DZ twin pairs. The difference in BMI means and variances between MZ and DZ twin pairs were not statistically significant, thus the equal means and variances assumption was not violated. It is noteworthy that both the BMI mean and variance was higher for singletons than for twins. This discrepancy has also been found for phenotypes such as body size and muscle strength and has previously been thoroughly discussed [23].

When analysing the twin data, the ACE model provided at marginally better fit of the data than the ADE model. The standardized A, C and E components were estimated at 0.80 [95% CI 0.73–0.84], 0.02 [95% CI 0.02–0.03] and 0.18 [95% CI 0.18–0.19], respectively. The ACE model was compared to the AE model. With a chi-square difference of 0.385 and a difference in degrees of freedom of 1, the p-value was 0.535 indicating that the ACE model did not fit the data better than the more parsimonious AE model. Hence, evidence for the contribution of common environmental influences to BMI variation was not found in the current data. The standardized A and E components were estimated at 0.82 [95% CI 0.82–0.84] and 0.18 [95% CI 0.18–0.19], respectively, under the AE model. Since the AE model will be used, we will, from now on, simply refer to the additive genetic variance as genetic variance.

Among full-brothers the total BMI variance increased from 5.7 in the 1951 birth cohort to 9.9 in the 1983 birth cohort. The BMI covariance increased in a similar proportion from 2.1 to 3.9 within the period studied. The correlation coefficients for BMI within the brother pairs increased slightly from around .375 [95% CI .340–.410] to .394 [95% CI .345–441], which translates into an increase in BMI heritability from 75% to 78.8%, although not statistically significant.

**Figure 2:**
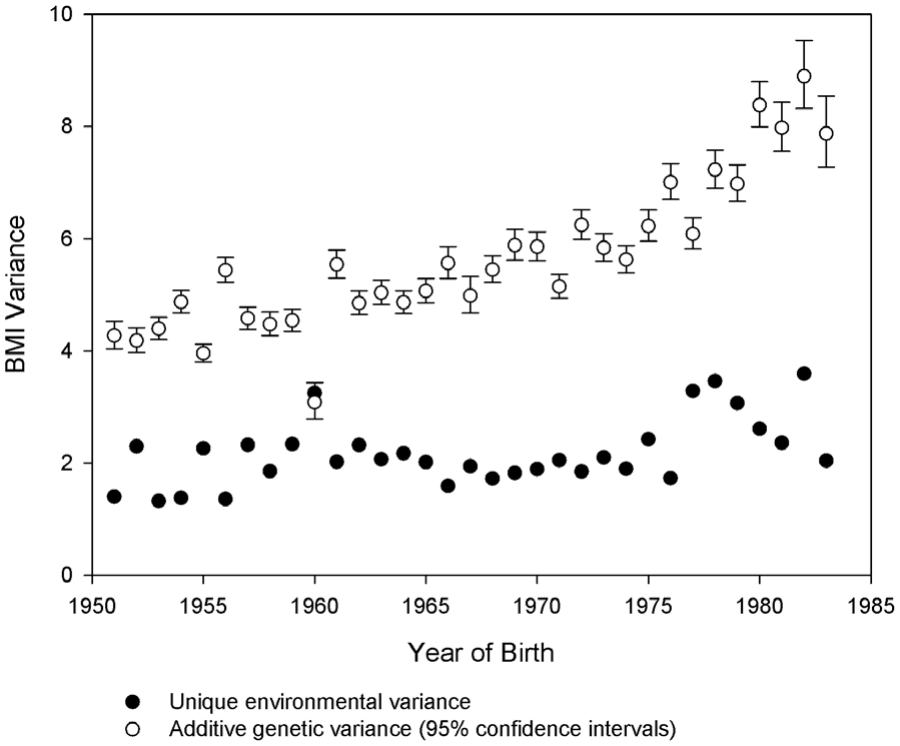
The additive genetic variance (95% confidence intervals) and unique environmental variance of BMI plotted against year of birth.

Since we can assume an AE model the genetic variance is twice the covariance within full-brother pairs. Hence, the genetic variance increased from 4.3 [95% CI 4.04–4.53] to 7.9 [95% CI 7.28–8.54] over the period studied (Figure 2). The increase was moderate from 1951–73 where after the genetic variance increased strongly. The unique environmental variance is responsible for the remainder of phenotypic variance and hence increased from 1.4 [95% CI 1.32–1.48] to 2.0 [95% CI 1.89–2.22] within the period (Figure 2).

**Figure 3:**
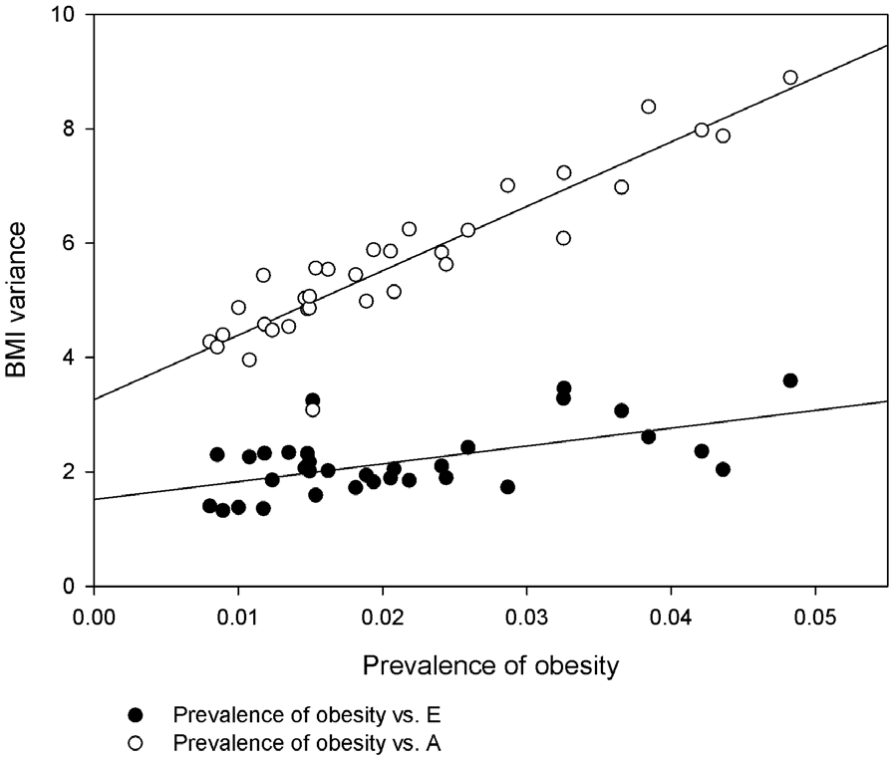
The prevalence of obesity plotted against the additive genetic and the unique environmental variance of BMI for each birth cohort. The regression lines are shown for the genetic and environmental variance vs. prevalence of obesity.

In Figure 3 the prevalence of obesity is plotted against the genetic and environmental variance for each birth cohort and regression lines are shown for both sources of variance. The slopes of both regressions were statistically significant (p<0.001 for both genetic and environmental variance). The Pearson correlation coefficient for the relationship between the genetic variance and prevalence of obesity showed a very high positive correlation of .921 [95% CI .844–.960], while the correlation between the environmental variance and prevalence of obesity was .572 [95% CI .285–.765].

The analyses were also conducted with the logarithm of BMI to test whether the same results would be obtained when the slight right skewness of the BMI distribution were taken into account. This transformation did not lead to different results and to ease the interpretation only the untransformed results are presented.

## Discussion

The results of structural equation modelling of MZ and DZ twins showed that a model including additive genetic and non-shared environmental factors adequately explains variance in BMI. These findings are also consistent with results from most adoption studies of non-biological siblings [24]. The analyses on birth cohort strata of full-brothers showed a statistically significant increase in BMI variance and covariance. The additive genetic variance of BMI increased strongly from 4.3 to 7.9 while the heritability of BMI increased slightly from 75% to 78.8%. The unique environmental variance of BMI showed a moderate increase from 1.4 to 2.0.

The results support the hypothesis that the genetic variance has increased in Sweden between 1969 and 2001. In parallel with the increasing prevalence of obesity, the increase in the genetic variance was moderate from the first observations in birth cohorts from 1951 until around 1973. From around birth cohort 1973 and onward the increase became stronger. The trend in the genetic variance was highly correlated (.921) with the trend for the prevalence of obesity in the same population, supporting the notion that it may be the obesogenic environment that drives the upward trend in the genetic variance (Figure 3). The close relationship points to a dose-response relationship between the exposure to the obesogenic environment and the genetic variance. Hence, the findings suggest GxE between the obesogenic environment and adiposity related genes in the direction of stronger genetic influences on adiposity in a more obesogenic environment.

The results show that the strong increase in BMI variance primarily is driven by the increase in genetic variance and only to a small extent by an increase in the environmental variance. This implies, that although the influence from the obesogenic environment has increased during the last decades [25], it is primarily the genetic background that creates the considerable variation in body fatness within populations and as the force of the obesogenic environment has become stronger the genetic differences have become more pronounced.

The increase in the genetic variance can be decomposed on basis of the mathematical formula for the contribution from one genetic locus which equals p(1-p)a^2^, where “p” denotes the population prevalence of an arbitrary obesity-related allele and “a” is the effect of that allele [26]. In the current study the population strata was defined by year of birth. This will most likely imply that the gene pool between strata is comparable, since, except for possible changes in genetic variation due to immigration, the gene pool does not change noteworthy within a few decades. This allows us to assume that “p” is constant across the birth cohorts. Hence, there are two main explanations to the increase in additive genetic variance in the more recent birth cohorts. Either the effect of loci already contributing to adiposity increases over time or new genes not previously expressed are activated as the influence from the obesogenic environment increases. Naturally, a combination of the two explanations is possible as well. Thus, the results indicate that the obesogenic environment modifies the expression of genes related to body fatness in the direction of stronger genetic influences in a more obesogenic environment.

The influence of the obesogenic environment on the genetic variance of BMI is possibly the result of GxE, which could occur in several ways. The genetic effects could be modified if the strength of an association in a biological pathway from genotype to phenotype is influenced by environmental circumstances. Furthermore, it can be argued that the causal pathway from genotype to phenotype goes beyond the limits of our physical organism. If a behavioural pattern mediating the genetic influence on obesity is either suppressed or facilitated under certain environmental conditions, it is reasonable to assume that the strength of an association between genotype and phenotype will change. Other types of mediating phenotypes under genetic influence could play a role, e.g. that an individual's perceived level of stress is under genetic influence and that, in turn, stress is a predictor of body fatness. If the environment changes and becomes more stressful it will have the strongest impact on individuals with a genetic susceptibility to stress. The association between genotype and BMI would then increase and thereby contribute to genetic variation in BMI. Finally, the GxE may involve a combination of different mechanisms.

This study is primarily exploratory and currently we have very limited knowledge concerning which mechanisms and environmental factors are actually underlying the findings. Considering the magnitude of change in genetic variance in a more obesogenic environment, the findings strongly encourage research in how the environmental influences alter the genetic contribution to differences in BMI, which would possibly allow a better targeting of preventive efforts in the future.

The results align with a recent study using quintile regression of genetic associations with BMI in children [27]. The authors calculated a genetic score based on five previously confirmed genetic markers of obesity. The genetic markers showed stronger associations with BMI in the higher BMI quintiles. Although the genetic profile may not be similar between the quintiles it complements the current findings by showing stronger genetic effects on adiposity among subgroups, which may be more exposed to the obesogenic environment. These results may be a consequence of GxE and/or gene-gene interaction, and the latter may be the case if the gene pool differs between the quintiles.

Several studies have examined whether putative risk factors for obesity modify the genetic variation in BMI. Although the direction of causation between obesity and physical activity is unclear, the level of physical activity may still be an indicator of the exposure to the obesogenic environment. Hence, the consistent evidence for an interaction between physical activity and the additive genetic variance in BMI, where higher genetic variance and stronger candidate gene effects are found in groups with a lower physical activity, points in the same direction as the current results [9]–[15]. The same argument applies to a recent study on Danish twins where a lower genetic variance in BMI was found with a higher level of education, which is known to be inversely associated with obesity [28]. In addition, it is worth noting that common variation in the *CHRNA5-CHRNA3-CHRNB4* gene region (chromosome 15q25) is associated with BMI in ever smokers, but not in non-smokers, which implies that also smoking modifies the genetic variation in BMI [29]. However, an important strength in the current study is that the population strata are defined by birth year, rendering the gene pool comparable across strata. Hence, the changes in genetic variation can be attributed to environmental modification of genetic effects rather than differences in the gene pool between strata.

In addition to possible confounding from the C and D components, assortative mating may play a role as well. The assumption of equality of gene frequencies across birth cohorts relies on the assumption that mating occurs at random [20]. If, on the other hand, mating occurs more frequently among individuals with genetic similarity, the genotype frequency could change across birth cohorts. This would undermine our interpretation that the increase in genetic variance could only be the result of changes in the genotypic effects. However, assortative mating is unlikely to account for the strong increase in genetic variance. It occurs primarily in the highest percentiles of the BMI distribution and thereby presumably contributes only little to the general variation in BMI [30]. Furthermore, assortative mating would increase the similarity of DZ twins and thereby mimic common environmental influences. Thus, the non-significant C component in the twin study supports the assumption of no assortative mating.

An important strength of the current study is that all participants were measured at approximately the same age, i.e. around 18–19 y and through the use of standardized measurement techniques. The genetic influence on adiposity may be age-dependent and measurement error is modelled as unique environmental variance, which attenuates the estimate of heritability. Hence, keeping these factors nearly constant allows a more accurate calculation of the genetic contribution to BMI. Furthermore, the collection of data from mandatory conscription examinations implies that the cohort is not self-selected, but is highly representative of the general population.

Another strength is that this study utilizes the impressive size of the Swedish conscript database. The classical twin design would tend to suffer from statistical power problems in addressing the secular trend in variance components. For example, the current population of nearly 1.5 million conscripts included merely 3,699 twin pairs eligible for analysis. Since the AE model proves to be the most parsimonious model for decomposing BMI variance, the analysis of covariance in full-brother pairs is by far the most statistically powerful approach to study secular changes in the variance components of BMI.

The study is subject to various limitations. Using full-brothers instead of twins comes at a price. While twin pairs are born at the same time full-brother pairs can be years apart. Although we limit our analyses to pairs with a maximum age difference of 3 years, the obesogenic environment may still change slightly between the births of two brothers. In periods with rapid secular changes in the obesogenic environment, this may influence the correlation and covariance estimates and hence bias the results. However, it seems unlikely that such biases could alone produce the relatively uniform secular trends and would probably rather tend to dilute the results. Likewise the twin analyses may have produced biased estimates of heritability. Perhaps the most critical assumption is the ‘equal environments assumption’, implying that MZ twins do not share a more similar environment than DZ twins [20]. This assumption may however not always be valid, e.g. MZ twins may be treated more similarly by their surroundings than DZ twins [31]. This could potentially increase their phenotypic similarity later on and in turn generate inflated estimates of heritability. Similarly, if MZ twins are more similar than DZ twins in terms of epigenetic characteristics [32] the heritability will also tend to be inflated. However, this problem is not of serious concern in the current analyses since the twin pairs are used primarily to test the significance of the common environmental influence. Furthermore, our primary interest is not in the absolute size of genetic influence but rather in investigating secular changes herein. Another limitation is that some DZ twins may falsely be classified as MZ twins and vice versa. However, with a relatively low misclassification rate (less than 4%) studies have confirmed that questionnaires are a valid method of zygosity determination [33]. Hence, this issue should not bias our results considerably. Finally it should be noted that the analyses only include males. Corresponding analyses would have to be carried out in female sibling pairs before we can determine whether the findings are representative of the entire population.

The findings may have both theoretical and practical perspectives. Firstly, they may elucidate the mechanisms of GxE during the last decades and thereby improve our general understanding of the role of adiposity-related genes in the obesity epidemic. It may also nuance our understanding of genetic factors as, rather than being fixed from conception, are quite flexible units in terms of their influence on phenotypes. Secondly, the findings may have a practical relevance if they are used instrumentally in genome wide association studies (GWAS). Since the total genetic variation is much higher in populations under a strong influence from the obesogenic environment, the GWAS samples may be selected to yield the highest genetic variance thereby improving the chances of finding statistically significant genetic effects. Furthermore, since the increase in genetic variance could be explained both by an increase in the influence from genes as well as by the activation of novel gene effects, this approach may also allow the discovery of new adiposity related genes.

In summary, the twin analyses showed that a model including additive genetic and unique environmental variance adequately explains BMI variance. On basis of the AE model the variance and covariance of BMI within Swedish brother pairs confirmed the hypothesis that the genetic variance in BMI has increased strongly throughout the obesity epidemic, suggesting GxE. The influence of genes can potentially be modified by the environment in any part in the causal pathway linking genotype to phenotype, including the restriction of facilitation of behavioural patterns related to obesity. The findings may have both theoretical and practical perspectives. Firstly, they may be a step towards a better understanding of the general role of genes during the obesity epidemic. Secondly, the findings could be used instrumentally in GWAS studies. It is plausible that if study population with the highest genetic variance is selected the chance of finding new genetic variants is improved. Thirdly, the results strongly encourage research in how the environmental influences alter the genetic contribution to differences in BMI, which would possibly facilitate development of better targeted preventive efforts in the future.
